# Integrating knowledge graphs with ancient Chinese medicine classics: challenges and future prospects of multi-agent system convergence

**DOI:** 10.1186/s13020-025-01226-7

**Published:** 2025-10-07

**Authors:** Shate Xiang, Huanxiang Lin, Fen Cai, Zhehan Jiang

**Affiliations:** 1 Institute of Medical Education Healthcare Science Center, Peking University, Beijing, 100083 China; 2 Chaoxing Joint Laboratory for Digital and Smart Medical Education, Peking University Health Science Center, Beijing, 100083 China

**Keywords:** Ancient Chinese medicine classics, Knowledge graphs, Multi-Agent systems, Applications

## Abstract

The inheritance of knowledge from Ancient Chinese Medicine Classics (ACMC) confronts challenges including fragmented literature, terminological heterogeneity, and reliance on traditional apprenticeship. Knowledge Graphs (KG) have become one of the tools for the digitalization and intelligentization of ACMC, playing a vital role in unifying terminology, standardizing data, and structuring and linking knowledge. However, due to the complexity of the ancient Chinese language in ACMC texts and the diversity of syndrome differentiation systems, current KG construction techniques still rely on manual input or traditional Natural Language Processing, with applications primarily limited to basic question-answering (Q&A) systems. Although large language models (LLMs) in the field of traditional Chinese medicine have incorporated ACMC corpora, automated extraction and intelligent integration within KG remain underdeveloped. This paper proposes an innovative approach that combines Multi-Agent Systems (MAS) with KG for advancing the intelligent application of ACMC. The technical approach involves using KG as the knowledge foundation, while leveraging MAS’s LLM-based semantic understanding and collaborative task distribution to enable breakthroughs in triple extraction technology and to advance the intelligent applications of ACMC, including context-aware Q&A, herbal formula innovation, dynamic diagnosis and treatment, and personalized education. Additionally, the integration of Retrieval-Augmented Generation technology enables the dynamic synthesis of multi-source knowledge, resolves semantic ambiguities, and optimizes MAS decision-making. These discussions aim to inform the design of a high-fidelity, adaptive, and perception-driven autonomous system for the intelligent inheritance and innovation of ACMC.

## Background

Traditional Chinese Medicine (TCM), as the original medicine of Chinese national science, demonstrates tremendous medical value globally through its unique philosophy of syndrome differentiation and treatment as well as its rich clinical experience [1]. The academic system of TCM is vast and comprehensive, with ancient Chinese medicine classics (ACMC) serving as the foundation of its theoretical framework and the cornerstone for cultivating clinical thinking [2]. ACMC include *The Yellow Emperor’s Classic of Medicine*, *Treatise on Cold Damage*, *Synopsis of Prescriptions of the Golden Chamber*, *Item Differentiation of Warm Febrile Diseases*, among others.

ACMC have played a pivotal role in the development of TCM. For instance, *The Yellow Emperor’s Classic of Medicine*, written between the Warring States period and the Western Han period in China, is the earliest surviving medical theory text in China. It established the theoretical system of TCM and construct the theoretical framework for its theory. Chinese doctor Zhang Zhongjing wrote *Treatise on Cold Damage* and *Synopsis of Prescriptions of the Golden Chamber*, connected theory with clinical practice and introduced the “Syndrome differentiation of six meridians” system, which became the cornerstone of TCM’ s unique approach to diagnosis and treatment. Additionally, *Item Differentiation of Warm Febrile Diseases*, authored by the physician Wu Jutong, established the “Wei-Qi-Ying-Blood” theory and the “Tri-Jiao Theory” method for warm febrile diseases, providing a systematic theoretical framework for their diagnosis and treatment.

It is evident that ACMC forms the cornerstone of the inheritance and development of TCM, providing crucial guidance for clinical practice. At the same time, it carries the ancient cultural essence of the Chinese nation and is an indispensable part of world culture. Therefore, the inheritance and development of ACMC is of utmost importance.

However, the inheritance of ACMC currently faces multiple challenges. On one hand, the inheritance of ACMC knowledge is still predominantly dependent on the traditional “master-apprentice” model, whose inherently private and individualized nature limits the broad dissemination and systematic construction of knowledge frameworks  [3]. On the other hand, independent learners are often confronted with fragmented and scattered literature resources that lack structural coherence, thereby raising the overall learning threshold  [4]. In addition, the pace of theoretical innovation and practical advancement within the TCM system based on ACMC remains relatively slow, leading to insufficient momentum for sustainable development. Therefore, it is imperative to explore effective strategies to overcome the dual obstacles of ACMC inheritance and TCM innovation.

With the rapid development of artificial intelligence (AI), there seems to be a technical solution to this issue [5]. Among them, knowledge graphs (KG), as a powerful tool, have been widely applied in the inheritance and dissemination of knowledge across various fields. It is also considered an effective means to enhance the utilization of classical Chinese medicine texts, addressing issues such as limited data applicability and management deficiencies [6]. For instance, the KG of ancient TCM book constructed by Yang Zhou [4] showcases the core concepts of TCM, including diseases, symptoms, prescriptions, medicines, and related dimensions such as regions, doctors, and dynasties. Similarly, the KG of TCM health preservation designed by Tong Yu [7] has also gained significant recognition in practical applications.

In addition, KG offer advantages in promoting the deep integration and efficient utilization of multi-source medical knowledge [8]. By deeply integrating with various AI technologies, KG open up vast prospects for intelligent applications of knowledge, including the development of knowledge-based question and answering (Q&A) systems, improved information retrieval efficiency, personalized recommendation services, and intelligent diagnosis and treatment [9, 10]. Currently, with the rise of multi-agent systems (MAS), the integration of KG and MAS has undoubtedly become a new opportunity to advance the intelligent development of knowledge and medicine [11]. Unfortunately, in the field of ACMC, despite initial explorations into building KG for classic texts such as *Treatise on Cold Damage* and *The Yellow Emperor’s Classic of Medicine*, as well as intelligent Q&A systems [12, 13], further application research has progressed slowly. Moreover, the integration of KG and MAS remains a largely unexplored area, requiring deeper investigation and expansion.

In view of this, this paper attempts to summarize the current state and limitations of KG applications in the field of ACMC. Building upon this, it incorporates the latest research findings on MAS to explore in depth the future application trends and potential challenges of integrating ACMC with these two technologies. We aim to provide valuable references and guidance for the development, inheritance, and practical application of intelligent ACMC.

## Application and impact of KG in the field of ACMC

### The concept of KG

KG is a graph data structures that focuses on named entities, concepts, and their relationships. It uses “triplets” as the basic unit, where entities are nodes, relationships are the edges connecting the nodes, and attributes describe the characteristics or states of the entities [14]. With its powerful semantic processing and open interconnectivity, KG can effectively integrate various types of knowledge, forming a complete and organized knowledge system. This structure not only enables the visualization of knowledge as a network graph but also significantly improves the efficiency of knowledge indexing, processing, and retrieval [15].

The concept of KG originated in the 1950s, with the introduction of the semantic network idea by M. Ross Quillian in 1968, followed by the rise of ontology research. The launch of the Google Knowledge Graph in 2012 marked the point at which KG entered mainstream applications [16]. KG integrates the concepts of semantic networks and ontologies, facilitating the exchange and processing of knowledge both between computers and between humans and machines. At present, KG have become a key technology in AI and are widely used in fields such as healthcare, finance, social media, and education. The construction of a KG involves several steps, including data collection, entity recognition, relationship extraction, knowledge integration, and storage [17]. Its functions encompass intelligent search, question answering, recommendation, and relationship analysis [18].

### Application of KG in the field of ACMC

As a robust knowledge representation framework, KG have demonstrated significant applicability in the domain of ACMC. The extensive corpus of ACMC includes four foundational texts—*The Yellow Emperor’s Inner Canon, Treatise on Cold Damage, Synopsis of Prescriptions of the Golden Chamber, and Item Differentiation of Warm Febrile Diseases*—collectively designated as the “Four Classics of TCM”. These works constitute the theoretical and clinical bedrock of Chinese medicine, having undergone centuries of rigorous scholarly engagement. They now serve as core pedagogical instruments in TCM education and central assessment metrics for classical competency in national examinations [19, 20]. Accordingly, these four texts have become focal points of both academic research and practical application. A review of the existing literature reveals that the integration of KG with the four canonical texts primarily focuses on the construction of knowledge theory and the practical application of technology.

Firstly, in terms of knowledge theory, Qianqian Li [21] designed and manually annotated a KG for *Treatise on Cold Damage* based on the diagnostic and treatment thinking model of *Disease and Pulse Syndrome and Treatment*, demonstrating the structural and logical relationships between its key concepts. Feng Yang [22] provided a detailed description of a manual annotation scheme for knowledge elements in *Treatise on Febrile and Miscellaneous Diseases*, aiming to improve the efficiency of computer-assisted knowledge extraction from classical texts. Yizhuo Zhang [23] employed the ontology seven-step method to construct an ontology for *Treatise on Cold Damage*, offering a conceptual model and logical foundation for KG construction, as well as templates and standards for knowledge extraction. These studies lay the groundwork for subsequent intelligent applications.

Secondly, in terms of technical application, Haodan Kuang [24] built a KG for *Treatise on Cold Damage* based on an ontology design. After standardizing the text through manual annotation and other processing steps, they utilized the Neo4j graph database to construct the KG. Additionally, the study developed the TCMKGLS-CRF model, which was applied to identify literature on the *Yangming Disease* from the works of fourteen medical practitioners spanning the Song to Qing dynasties. Qianqian Qu [25] conducted an in-depth study of *Treatise on Cold Damage* based on Natural Language Processing (NLP) techniques, comparing the effectiveness of different neural network models for entity recognition. They selected the optimal model and constructed a KG focused on “Symptoms-Formulas-Medicine”, enabling the querying and analysis of treatment patterns for liver-related symptoms, formulas, and drugs within *Treatise on Cold Damage*. Dongbo Liu [12] employed various NLP methods for knowledge extraction to build the KG of *Treatise on Cold Damage*. Jingwei Wang [26] further developed an intelligent Q&A system based on KG for *Treatise on Cold Damage*. This system utilizes KG and NLP technologies to quickly retrieve fundamental knowledge from *Treatise on Cold Damage*, stores the data in the Neo4j graph database, and uses techniques such as multinomial naive Bayes models to automatically map questions to answers. Furthermore, Longxiang Chu [27] extended the KG of *Treatise on Cold Damage* to research a personalized recommendation model for course learning resources. This model facilitates interaction and collaboration among learners and provides precise, personalized recommendations for learning materials based on users’ learning needs, historical records, and progress.

In addition, Yecheng Feng [13] also utilized NLP techniques for knowledge extraction from *The Yellow Emperor’s Classic of Medicine*. Through steps such as text preprocessing, implicit relationship extraction, and knowledge system visualization, they successfully constructed a KG tailored specifically to this classic text. Xiaoxiao Zhang [28] built a KG for *Synopsis of Prescriptions of the Golden Chamber* and integrated it with the diagnostic and therapeutic patterns for postpartum abdominal pain, enabling the visualization of clinical knowledge and further enhancing the practical value of the KG. Xiaolan Zou [29] constructed a knowledge graph of *Warm Disease Theory*, which encompasses classical texts of the Warm Disease school (including *Item Differentiation of Warm Febrile Diseases* [30]), and developed a KG-based question answering (Q&A) system.

**Fig. 1:**
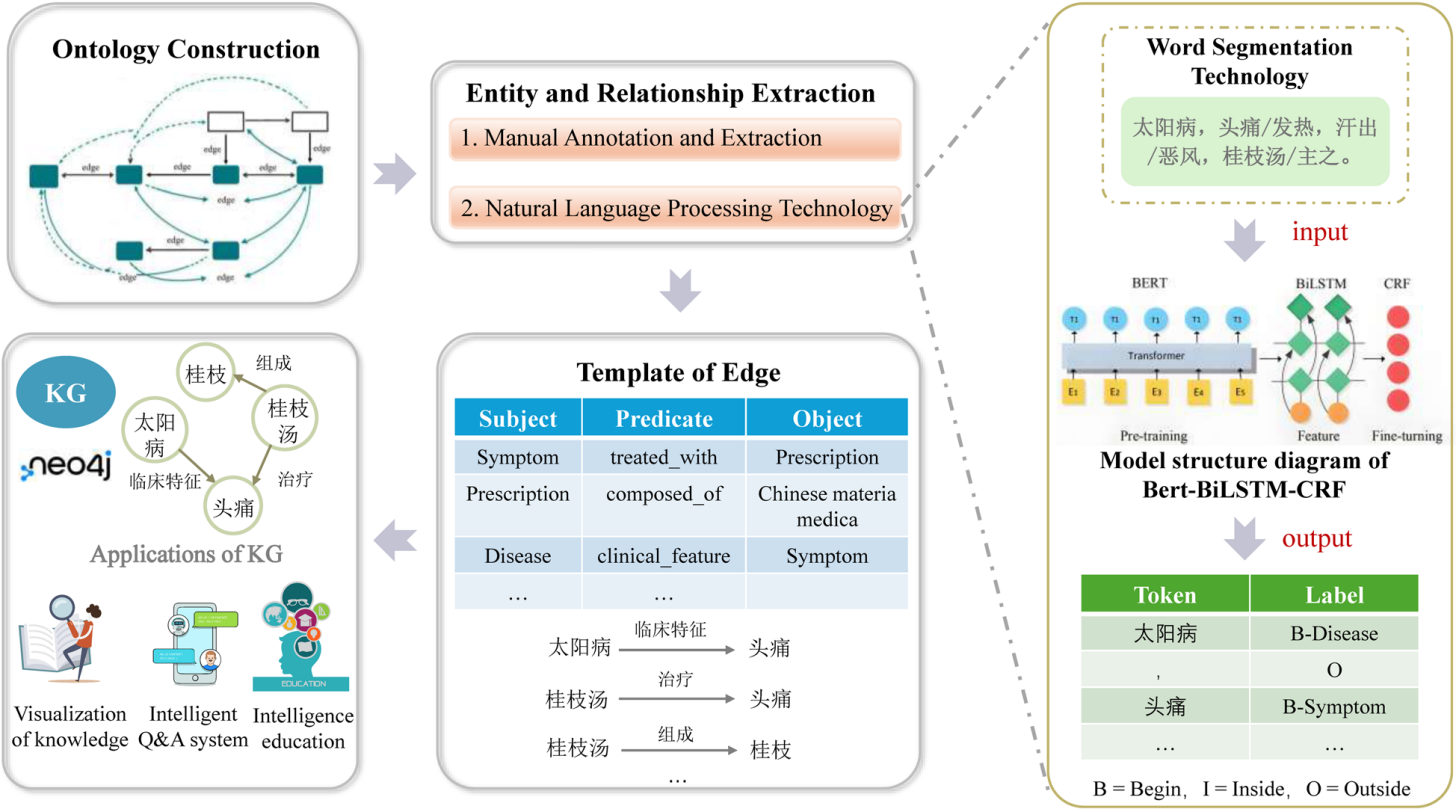
Sketch of the construction and application of KG of ACMC

The technical methods related to the studies mentioned above are shown in Table 1. Through an analysis and synthesis of the methods of KG construction in the literature (Fig. 1), we can observe that, so far, the construction of KG for the “Four Classics of TCM” primarily involves three main steps: ontology development, entity and relationship extraction, and knowledge storage and visualization. In terms of entity and relationship extraction, there are two approaches: manual annotation and NLP techniques, with variations in the NLP technologies used across different studies. This indicates that AI-based named entity recognition technologic is still in the process of continuous exploration and development. Additionally, the application of KG in ACMC has achieved the systematization of knowledge, intelligent Q&A, and personalized learning material recommendations, but there remains significant untapped potential for further exploration.

**Table 1:** Overview of KG construction strategies and outcomes in the “Four classics of TCM”

Study	Research methodology	Research results
Haodan Kuang [24] (2021)	Ontology construction: The ontology was built by drawing on the theoretical framework of TCM knowledge engineering KG construction: Named entity annotation was (using a specialized knowledge service system for Chinese medicinal herbs) marked the original text and standardized the relationships between various concepts. The data was then imported into the Neo4j graph database to construct the KG Named entity recognition: A comparative study was conducted on the effectiveness of CRF, BiLSTM, pretrained BiLSTM, and TCMKGLSTM-CRF models for entity recognition. The TCMKGLSTM-CRF model was ultimately chosen to extract entities from fourteen works analyzing Yangming disease in *Treatise on Cold Damage* KG updating and expansion: New knowledge triplets (such as treatment methods, medicines, etc.) obtained through named entity recognition technology were integrated into the existing KG for further expansion	The KG of *Treatise on Cold Damage* was successfully constructed, and based on this, new methods for treating Yangming disease introduced by renowned physicians from the Song to Qing dynasties were further expanded and supplemented
Qianqian Qu [25] (2021)	Word segmentation technology: A comparison was made between dictionary-based, machine learning, and deep learning word segmentation methods, and the optimal approach was selected Word vector construction: Word vectors were constructed using word2vec to effectively represent the vocabulary in Treatise on Cold Damage Named entity recognition: By comparing three models—word2vec-BiLSTM, word2vec-BiLSTM-CRF, and Bert-BiLSTM-CRF—the Bert-BiLSTM-CRF model was chosen for named entity recognition to identify entities in *Treatise on Cold Damage* KG construction: The extracted entities and entity relationships were imported into The Neo4j graph database to construct the knowledge graph	A named entity recognition system for *Treatise on Cold Damage* was successfully constructed, capable of accurately identifying entities such as diseases, symptoms, and medicine
Dongbo Liu [12] (2022)	Ontology construction: The seven-step method was used to construct an ontology for the field of TCM. Through in-depth analysis of core concepts in *Treatise on Cold Damage*, such as diseases, syndromes, symptoms, treatment methods, and prescriptions, the conceptual hierarchy and interrelationships were defined Knowledge extraction: The NLPIR-ICTCLAS Chinese word segmentation system was used to perform Chinese word segmentation and extract entities related to diseases, syndrome, symptoms, methods, and prescriptions Knowledge integration: A combination of rule-based and statistical methods was used to align entities, and expert review methods were applied to complete the knowledge integration KG construction: The Neo4j graph database was utilized to store and manage the entity relationship network. With the powerful capabilities of the graph database, efficient knowledge retrieval and visual display were achieved	The “disease-syndrome-symptom-treatment-prescription” KG for *Treatise on Cold Damage* was successfully constructed
Jingwei Wang [26] (2022)	KG construction: A rule-based approach was used to extract entities and their relationships, and the Neo4j graph database was employed for storage Named entity recognition: A BiLSTM + CRF model was used, with experiments conducted using different embedding layers (word2vec, BERT, ALBERT), and ALBERT was selected as the optimal model Relationship classification: A multinomial naive Bayes model was used to train the relationship classification model Q&A system design: Cypher query templates were designed to enable automatic mapping from questions to answers	The KG of *Treatise on Cold Damage* was successfully constructed, and an intelligent Q&A system based on the KG was developed, capable of quickly and accurately answering user queries
Longxiang Chu [27] (2024)	KG construction: Building upon the preliminary KG research for *Treatise on Cold Damage*, a KG for the course was constructed by integrating the course ontology. Entities were extracted from the textbook, semantic relationships were established, and the data layer was completed Hybrid recommendation algorithm design: A hybrid recommendation algorithm was designed by combining collaborative filtering and path-based recommendation algorithms. Learning resources were recommended and ranked based on similarity Experimental validation: Learning materials for *Treatise on Cold Damage*, were collected, including images, texts, and videos. Data preprocessing, feature extraction, and simulated recommendations were carried out	A personalized recommendation model based on the course KG of *Treatise on Cold Damage* was successfully constructed, providing learners with more targeted and diverse learning resources for the course
Yecheng Feng [13] (2023)	Text preprocessing: Constructivist grammar theory was introduced, and a classification tree model was used to perform text classification. An entity vocabulary for *The Yellow Emperor’s Classic of Medicine* was built, and word segmentation, part-of-speech tagging, and entity annotation were completed Implicit relationship extraction: Syntactic trees were generated based on dependency syntax analysis, extraction rules for implicit relationships were defined, and algorithms were designed to validate their effectiveness Knowledge visualization: The Neo4j graph database was used for knowledge storage and visualization	A knowledge extraction method tailored for *The Yellow Emperor’s Classic of Medicine* was successfully constructed
Xiaoxiao Zhang [28] (2023)	Ontology construction: The domain ontology was constructed based on the seven-step method, defining the concept categories and interrelationships in *Synopsis of Prescriptions of the Golden Chamber* Entity extraction: The text entity recognition tool of Baidu Brain EasyData was used for manual annotation of the disease text set and treatment text set KG construction: The extracted entities and relationships were used to construct the KG, which was stored in the Neo4j graph database Data mining: An improved version of the Apriori algorithm and other association rule algorithms were used to mine the diagnostic and treatment patterns for postpartum abdominal pain from the *Great Dictionary of Chinese Medicinal Formulas* Knowledge integration and visualization: The diagnostic and treatment patterns for postpartum abdominal pain were integrated with the KG, enabling the visualization of clinical knowledge	The KG of *Synopsis of Prescriptions of the Golden Chamber* was successfully constructed, and the diagnostic and treatment system for postpartum abdominal pain was further incorporated into the application of the KG, enhancing its practical value
Xiaolan Zou [29] (2023)	Knowledge modeling: Thirty classical texts of the Warm Disease Theory were categorized into three core themes: Chinese materia medica, prescriptions, and medical theories Entity extraction: Named entities were annotated using Doccano, and extracted through rule- and dictionary-based methods implemented with HanLP (A natural language processing toolkit), followed by manual validation Entity alignment and relation extraction: The extracted entities were semantically aligned and normalized. Both explicit and implicit relationships among entities were identified and extracted KG construction: The extracted entities and relationships were used to construct the KG, which was stored in the Neo4j graph database System development: A knowledge-based question answering and visual retrieval system was developed based on the Neo4j database	Successfully constructed the KG of Warm Disease Theory and developed a graph-based Q&A system

### Impact of KG on the study of ACMC

Traditional research on ACMC primarily relies on literature collation, theoretical induction, experiential transmission, and clinical practice. However, these approaches often suffer from low efficiency, strong subjectivity, and fragmented information, making it difficult to support the systematic inheritance and intelligent mining of knowledge. With the advancement of KG technologies, their integration with the ACMC is opening new avenues for Chinese herbal medicine discovery, disease analysis, and the inheritance and development of knowledge.

#### Impact on Chinese herbal medicine research

During the construction of a KG, terminology normalization and data standardization are essential for improving the quality, consistency, and scalability of the graph [31]. In the process of entity recognition and relation extraction for ACMC, professional thesauri are often employed to unify terminologies. This enhances data coherence and system interoperability while ensuring that core concepts—such as prescriptions, Chinese herbal medicines, syndromes, symptoms, and diseases—are represented in a structured and semantically accurate manner [32]. For instance, the term “*Yinhua*” mentioned in *Item Differentiation of Warm Febrile Diseases* is a common alias for “*Jinyinhua*” (*honeysuckle*). Alias merging in this case can effectively reduce entity recognition ambiguity and semantic redundancy. Such standardization is critical to addressing the fragmentation and unstructured nature of herbal information in classical TCM formulas, thereby providing a high-quality knowledge foundation for downstream pharmacological research and semantic computation.

Secondly, KG support the development of semantically driven, interactive human–computer retrieval modules. By visualizing entities and their relationships, KG enable the construction of multi-level and multi-dimensional knowledge pathways and logical structures [33]. Researchers can intuitively query, explore, and learn about upstream and downstream associations of specific Chinese herbal medicine via semantic linkages within the graph. For example, through the path “*Ephedra → Component of → Ephedra Decoction → Treats → Wind-Cold Exterior Excess Pattern → Symptoms → Fever, aversion to cold, absence of sweating*”, one can quickly comprehend the contextual use and logical relationships of the Chinese herbal medicine within the framework of syndrome differentiation and treatment. This mechanism enhances the interpretability and accessibility of clinical medication practices and provides both data support and theoretical foundations for pharmacological research.

Moreover, several studies have constructed KG by integrating heterogeneous databases to establish deep connections between Chinese herbal medicine and modern biomedicine [34, 35]. These efforts particularly focus on linking herbal active compounds with their molecular targets, thereby promoting the integration of Chinese herbal medicine with modern medicine at both molecular and phenotypic levels. A representative case is Artemisia annua, whose active compound, artemisinin, interacts with targets such as MMP9 and ALB to regulate pathways involved in Plasmodium proliferation, thus exerting significant antimalarial effects [35]. This process not only facilitates the rediscovery of TCM knowledge but also enables the systematic reconstruction of its theoretical framework within a modern scientific context, providing strong support for the modernization and application of classical herbal formulas.

#### Impact on disease research

Disease-related descriptions are often scattered across different chapters or texts and expressed using varying terminologies and formats in ACMC, leading to fragmented information and challenges in standardization [36]. For instance, the indications of *Cinnamomi Ramulus Decoction* are dispersed across multiple clauses, requiring physicians to integrate them based on personal clinical experience. By constructing a KG, researchers can computationally integrate the “disease-pulse diagnosis-syndrome pattern-treatment” framework, enabling systematic structuring and standardization of clinical knowledge [32]. This approach facilitates the digital storage of disease-related content in ACMC and promotes the efficient transmission of medical knowledge.

KG can also dynamically illustrate the progression of a disease from onset to resolution. For example, they can demonstrate the transformation from syndromes to corresponding treatment strategies, and clearly map key symptoms, pathogenesis shifts, and potential therapeutic regimens at each stage. The KG developed by Zhou et al. [37] computationally integrates and visually renders the core pathogenesis, characteristic symptom profiles, Six-Meridian disease progression pathways, and evidence-based herb modification protocols associated with *Cinnamomi Ramulus Decoction*. Such visualized, evolution-oriented graphs facilitate a deeper understanding of disease mechanisms, support the discovery of syndrome-treatment patterns, and provide practical guidance for modern clinical applications.

By integrating multi-source data such as historical medical texts and clinical case records, KG can further elucidate latent disease regularities and reconstruct the evolution of therapeutic paradigms [38]. For example, Haodan Kuang [24] constructed a cross-era diagnostic and therapeutic KG that integrates treatment strategies for Yangming Syndrome proposed by renowned physicians from the Song to Qing dynasties. This not only expands the academic connotation of the *Treatise on Cold Damage*, but also reveals the evolution of diagnostic and therapeutic paradigms under different historical contexts. Similarly, Xiaoxiao Zhang [28] developed a KG based on the *Synopsis of Prescriptions of the Golden Chamber* for postpartum abdominal pain, enabling the integration of empirical case prescriptions into a systematic diagnostic and therapeutic framework (Table 1). Furthermore, the introduction of KG facilitates precise semantic querying using Cypher language, allowing dynamic interpretation of core pattern differentiation principles such as “different treatments for the same disease” and “the same treatment for different diseases”, thereby revealing both the heterogeneity and commonality patterns in disease diagnosis and treatment [39]. Through graph-based knowledge representation and inference mechanisms, KG not only improve the efficiency of classical text interpretation but also provide a computable framework for theoretical innovation in TCM disease research.

#### Impact on the inheritance and development of ACMC

As discussed above, KG facilitate the standardized representation, digital storage, and retrieval of knowledge from ACMC by integrating data cleaning, annotation, and storage into a unified process. During the integration of classical texts from different historical periods, KG help to clarify the developmental trajectory of ACMC and enhance the intrinsic connections among classical medical knowledge sources [40]. Moreover, by incorporating specialized pattern-determination system, KG align classical prescriptions with modern clinical thinking, thereby supporting their contemporary application [28]. In addition, the semantic query capabilities of KG significantly simplify the retrieval of ACMC-related knowledge and improve knowledge utilization efficiency. Overall, KG reshape the mode of knowledge representation for ACMC through digital and structured approaches, while transforming knowledge acquisition pathways via natural language interaction, thereby significantly enhancing the preservation efficacy and contextual depth of ACMC transmission.

With the advancement of AI and knowledge engineering, KG have evolved from traditional knowledge repositories into intelligent platforms capable of reasoning and decision-making. In the domain of ACMC, KG not only enable the structured representation of terms, concepts, and their interrelationships, but also support clinical-contextual knowledge inference and the discovery of latent associations through the integration of logical rules, distributed representations, and neural network-based reasoning algorithms. For instance, the KG system developed by Haiyu Liu et al. inferred a novel therapeutic relation—namely, that *Gegen Qinlian Decoction* can be used to treat acute enteritis—based on semantic overlaps among symptoms, thereby extending the modern application boundaries of traditional formula-pattern correspondence systems [40].

Building upon this foundation, KG can further support a wide range of applications, including intelligent question answering, clinical decision support, and personalized education, thereby promoting the dynamic updating and intelligent utilization of ACMC knowledge. Although related research is still in its early stages, the demonstrated capacity of KG for knowledge organization and reasoning has already laid a solid foundation for the intelligent development of ACMC.

## Challenges in the application of KG in ACMC

### Limitations of intelligent entity and relationship extraction

Given the distinct historical characteristics of the language in ACMC: (1) the lack of a unified and standardized terminology system [41]; (2) the flexible and variable language structure, which does not adhere to fixed grammatical rules [42]; (3) the diverse writing techniques, including intertextuality, ellipsis, inversion, and other rhetorical strategies [43]; (4) the existence of synonyms in the description of symptoms, where different symptoms may share similar textual descriptions [44]; and (5) the significant entity nesting structure inherent in the TCM theoretical system [45], where high-level abstract concepts (such as the Five Elements and Yin-Yang) are intricately intertwined with concrete physiological entities (such as viscera and meridians).

These features make the content of ancient texts particularly obscure and difficult to understand. Moreover, they significantly increase the complexity of entity and relationship extraction in AI, hindering the automation of ontology construction and entity extraction for KG. As a result, these tasks still heavily rely on manual operations by specialists to ensure accuracy and deep understanding.

With the continuous development of AI, particularly the emergence of large language models (LLMs), research combining KG with AI has entered a new phase. The powerful semantic understanding and reasoning capabilities of LLMs enhance the intelligence and accuracy of entity and relationship recognition, enabling the extraction of a large number of “triplets” from unstructured text [46]. Researchers have also made valuable explorations in the unique field of TCM. For example, Yuhao He [47] effectively extracted entities and relationships from *Zhonghua Yifang* (a non-ACMC text) using the Chat Generative Pre-Trained Transformer (ChatGPT), achieving relatively accurate extraction results. Yichong Zhang [48] used the iFLYTEK Spark Cognitive Large Model to perform knowledge extraction from TCM texts and compared it with ChatGPT. The results showed an accuracy rate of 94.09%, outperforming the comparison model. Compared to traditional NLP methods, LLMs do not require the construction of complex specialized thesaurus or the writing of intricate programming, significantly simplifying the extraction process. They are also capable of automatically performing error detection and correction [49].

However, existing studies on information extraction in the TCM domain have primarily focused on general TCM texts, limiting the direct applicability of their findings to ACMC. There are two main reasons for this: First, the TCM texts included in current studies often possess a certain level of system logic, with a clear and organized structure of information. This characteristic helps LLMs more accurately identify and understand key information, concepts, and their interrelationships within the text, potentially leading to higher applicability and accuracy. Second, the output of LLMs is heavily dependent on the design of the prompts [50]. Prompts designed for works such as *Zhonghua Yifang* or broader TCM texts are not applicable to ACMC.

Given the specific characteristics of the ACMC, we further analyzed TCM-oriented LLMs that integrate ACMC training data, evaluating their strategies for processing and their entity recognition capabilities on the original ACMC texts. As summarized in Table 2, mainstream preprocessing approaches typically involve three key steps: (1) translating classical Chinese into modern Chinese using automated tools or LLMs; (2) applying character- and paragraph-level cleaning alongside structural normalization; and (3) employing generative LLMs via prompt engineering to refine translated content or generate natural language descriptions. This multi-step methodology aims to mitigate the semantic, syntactic, and structural complexity inherent in classical Chinese texts, thereby facilitating more effective information understanding and extraction by LLMs.

**Table 2:** Comparative analysis of core features in TCM-LLMs incorporating ACMC in training

LLM	Year	Preprocessing approach based on ACMC	Key features	Limitation
Qibo [51]	2024	Character-level cleaning rules, paragraph-level cleaning rules, and manually verified and iteratively refined	(1) Multi-turn dialogue-based diagnostic inquiry integrated with dynamic information retrieval from a TCM knowledge graph (2) Supports multiple tasks, including TCM prescription entity recognition, symptom classification, and machine reading comprehension	(1) Relies on manually annotated data (2) Understanding of classical Chinese requires human intervention (3) Lacks clinical expert validation of outputs
MedChatZH [52]	2024	Using Baidu’s translation tool to convert all classical Chinese materials into modern Chinese, then used the ChatGPT API to polish poor translations	(1) Multi-turn dialogue-based diagnostic inquiry integrated with dynamic information retrieval from a TCM knowledge graph (2) Focuses on TCM inquiry and health consultation	(1) Relies on translated data, introducing potential bias (2) Evaluation lacks real-world clinical validation
Huang-Di [53]	2024	Standardization of unstructured texts and integration of structured knowledge, combined with generative AI for instruction data construction and automatic quality optimization	(1) Supports interpretation of classical theories, terminology, and case records (2) Combined structured and unstructured corpus to enhance knowledge completeness	Limited ancient text parsing in ChatGPT may compromise data accuracy due to semantic misinterpretation
Lingdan [54]	2024	Classical texts were translated into modern Chinese using Baichuan2-13B-Chat, followed by data cleaning and structured storage. The structured dataset was then processed by ChatGPT-3.5 via prompt engineering to generate natural language descriptions	(1) Terminology normalization and structured storage, along with automated data cleaning and similarity-based integration, improved the quality of TCM data (2) Improve the precision and effectiveness of TCM-assisted diagnosis, treatment, and prescription	The translation of classical texts by Baichuan2-13B-Chat may not be fully reliable or accurate

Moreover, although some models have integrated KG to enhance their background knowledge, detailed information regarding the construction and utilization of these KG has not been publicly disclosed. To date, no published studies have explicitly investigated the direct use of these LLMs for entity extraction from ACMC texts. This observation suggests that current LLMs in TCM may still lack robust processing strategies and effective entity recognition mechanisms when dealing with the obscure language and loosely structured nature of classical medical texts, hindering deep semantic understanding and systematic knowledge acquisition at the level of the original classical Chinese.

In summary, the unique linguistic characteristics of ACMC increase the difficulty of entity and relationship extraction. Therefore, the application of LLMs in this field still requires targeted research and optimization. Developing an intelligent model to extract entities and relationships from ACMC texts and more accurately construct KG is a project that warrants further in-depth research.

### The lag in the applied research of KG in ACMC

In the field of non-ACMC fields, KG have gradually become a core foundation for the development of various intelligent applications, including knowledge reasoning, dynamic diagnosis and treatment, question generation, and adaptive testing. Specifically, modern medicine has constructed KG by integrating multi-source data (including information on diseases, medicine, medicine-disease relationships, chemicals and molecules, genes and proteins, biological pathways and networks, etc.), and using techniques like link prediction and graph convolutional networks, has achieved drug repurposing and adverse drug reaction prediction [55]. J. Liu [56] combined TCM knowledge with modern data to build a structured KG, and through serialization, gradually evolved patients’ conditions and treatment plans, creating a dynamic reasoning mechanism capable of suggesting appropriate treatments based on patients’ symptoms and signs. Furthermore, the entities and relationships within the KG can serve as fundamental materials for constructing questions. By combining advanced technologies such as semantic embedding, transfer learning, pre-trained language models, and semantic reasoning, the automatic generation of questions can be effectively supported [57]. Additionally, integrating KG with adaptive testing models can further explore and utilize the complex interrelationships between knowledge points, track and update the test-taker’s knowledge mastery in real time, thereby achieving precise question selection and dynamic adaptation [58].

It can be seen that although the construction of KG for the “Four Classics of TCM” has made initial progress in both theoretical and technical aspects, the pace of application-oriented research remains relatively behind when compared to non-ACMC fields. Given the close connection between the “Four Classics of TCM” and clinical practice, along with the detailed annotations and profound analyses by renowned physicians throughout history, their integration can greatly enhance knowledge density and improve knowledge reasoning capabilities. The valuable medical cases accumulated from ancient times to the present provide precious data support for constructing a “classical prescriptions” wisdom-based diagnostic and treatment system. The abundant research data on formulas and Chinese herbal medicines within ACMC, as explored by modern pharmacology, can furnish a multi-dimensional knowledge framework, thereby facilitating knowledge reconfiguration and innovation. Additionally, from an educational perspective, personalized learning and self-testing of ACMC play a crucial role. The development of a closely related testing system will undoubtedly greatly promote the learning effectiveness and in-depth understanding of learners, and help them better master the essence of TCM.

Although the application of KG for AMCM demonstrates immense development potential, it also faces a range of technical challenges and difficulties. First, the language of ACMC reference materials and texts (e.g., *Shanghan Laisu Ji*, *Shanghan Guanzhu Ji*, etc.) is complex, with descriptions involving numerous symbols, metaphors, and abstract concepts [59]. In addition, issues such as diverse writing standards, inconsistent terminology, and citation errors significantly increase the difficulty of natural language processing. However, current technological approaches still inadequate in handling this complex information. Secondly, the diversity of TCM clinical practices (e.g., acupuncture, herbal medicine) and personalized treatment characteristics place higher demands on the application of KG. TCM emphasize “syndromes differentiation treatment”, meaning personalized treatment plans are created based on the patient’s specific condition, constitution, and environmental factors [60]. This is especially true when using the “classical prescriptions” approach, which requires the construction of diagnostic thinking such as Six Meridians differentiation and Wei-qi-Ying-blood differentiation [61, 62]. Such a personalized treatment model and independent diagnostic thinking undoubtedly place high demands on the reasoning capabilities of KG, which may present a significant challenge in terms of technical implementation. Furthermore, when developing ACMC exam questions, the diversity and precision of differentiation in the question setting, the effective integration of authoritative TCM medical cases into the question generation system, and the hierarchical nature of the questions all pose high demands on the knowledge reasoning and integration capabilities of the KG system [63, 64].

## Introduction of MAS

The introduction of MAS brings new hope for the intelligent extraction and application expansion of KG in ACMC. In the face of challenges such as entity and relationship extraction, knowledge reasoning, and application, MAS, with its distributed, collaborative, and intelligent characteristics, holds the potential to enhance extraction accuracy, improve reasoning capabilities, and further extend to diversified applications such as intelligent Q&A and dynamic testing. Therefore, exploring the integration path of MAS and the KG of ACMC is crucial for the digital transformation and development of TCM.

### Overview of MAS

Agent is an autonomous entity capable of perceiving environmental inputs and taking actions through effectors to achieve goals. It is considered a flexible and self-reliant software component that provides interoperability interfaces for systems. MAS consists of multiple autonomous agents working together to simulate the collaborative behaviors of living organisms in nature. This enables the decomposition, parallel processing, and collaborative completion of complex tasks [65]. MAS offers various advantages, including task decomposition and parallel processing, collaboration and complementarity, flexibility and scalability, as well as robustness and reliability [66]. These benefits allow MAS to handle complex tasks, improve efficiency and speed of completion, while maintaining system stability and reliability.

So far, MAS has been widely applied in the field of modern medicine. For example, in cancer care, MAS integrates wireless sensor networks, body-area sensor networks, electronic health records, patient self-reports, and intelligent monitoring tools, enabling real-time collection, dynamic updating, and multi-party sharing of patient data, optimizing cancer management processes [67]. In the field of surgery, by integrating communication, updates, and specific application agents (such as agents controlling airway lasers, ventilators, and monitoring blood oxygen saturation), MAS has been used to create a system that facilitates information exchange and coordination among airway lasers, ventilators, and blood oxygen levels during airway laser surgery simulations, improving safety and efficiency [68]. In epidemiological research, combining GIS systems with real-time data, MAS uses fine-grained modeling of individual behavior and infection transmission to provide high-precision tools for epidemic prediction and policy formulation [69]. In immunology, researchers use MAS to virtually build agents for bacteria, immune cells, osteoblasts, and osteoclasts to simulate the early dynamics of human bone and joint infections, providing strong support for studying the pathogenesis of these infections [70]. The widespread application of MAS highlights its exceptional technical integration and data processing capabilities, bringing revolutionary breakthroughs to various fields of modern medicine.

Despite the numerous advantages of MAS, there are some shortcomings in its application within specific domains. Firstly, MAS tends to generate hallucinations, meaning it often produces unreal or inaccurate answers when tasked with handling problems beyond its knowledge and perception [71]. Secondly, MAS can suffer from heavy computational burdens and slow convergence in state exploration [72]. Lastly, MAS generally lacks a global knowledge representation framework, making it challenging for them to share and reason about knowledge on a global scale [73].

These limitations are also reflected in the application of MAS in the field of ACMC. The theoretical system of ACMC is vast and complex, encompassing concepts like Yin-Yang, Five Elements, viscera, and meridians, which significantly constrain the state and processing space of MAS. The inherent ambiguity and uncertainty of ACMC data runs counter to the need for MAS to have clear, standardized models and data inputs. Coupled with the fact that the combination of ACMC and modern technology is still in its infancy, the lack of mature technological frameworks and solutions has led to serious constraints on the application of MAS in the field of ACMC.

Therefore, to promote the application of MAS in the field of ACMC, it is essential to overcome its limitations and address issues related to the standardization, logic, and theoretical aspects of ACMC data. At the same time, it is necessary to explore effective ways to integrate AI technologies with ACMC knowledge, and to develop technical frameworks and solutions that are tailored to the unique characteristics of ACMC.

### Integration and application of MAS and KG

In order to overcome the above limitations, we consider the fusion of MAS with KG as a means of cracking. Currently, the fusion of the use of MAS and KG has emerged as an effective approach to address the challenges faced by MAS in specialized fields. As a structured knowledge representation method, KG provides MAS with rich, recognizable, and understandable information about concepts and their relationships, enabling the decisions made by the MAS to be firmly rooted in a comprehensive knowledge framework. This is crucial for guiding MAS in generating informed messages and making accurate decisions [74]. Upon reviewing the literature, we found that this integration has been applied in various areas, including intelligent Q&A, question generation, knowledge reasoning, and personalized recommendations.

In the area of Q&A systems, Jun Zhao [75] addressed the issues of hallucination and lack of accuracy of models by integrating MAS with KG. Their MAS design consists of three agents—Detector, Thought, and Answer—each responsible for tasks such as query error detection, generating inference analysis, and optimizing answers. This significantly improves the stability and accuracy of complex Q&A tasks. In terms of question generation, Runhao Zhao [76] proposed solutions for generating zero-shot KG in the absence of quality assurance data. The paper suggests combining LLMs with small model capabilities based on existing KG, forming question generation agents, proofreading agents, optimization agents, and coordination agents to efficiently and accurately generate questions. In terms of knowledge reasoning, Alireza Ghafarollahi [74] integrated experimental data, theoretical models, and literature from multiple disciplines to construct a KG, and designed an automated scientific development MAS. This MAS, consisting of agents responsible for experimental design, data analysis, hypothesis generation, and more, collaborates in a way that simulates the working mode of a scientific team, improving the efficiency and innovation of scientific research. In terms of personalized recommendations, Xujia Li [77] developed an MAS called Mcore, in which a coordination agent identifies users’ product category preferences, while relationship reasoning and entity recognition agents collaboratively perform KG reasoning to complete the product recommendation task.

These successful cases not only demonstrate the enormous potential of integrating MAS with KG in scientific research but also offer valuable insights for exploring new pathways for the intelligent inheritance of ACMC.

### Retrieval-augmented generation as a bridge between MAS and KG

Although MAS can enhance the efficiency and accuracy of task execution by leveraging structured knowledge from KG, their decision-making and task allocation processes often rely on predefined rules, making them less responsive to complex language inputs and dynamic contextual changes. Meanwhile, while KG offer systematic and structured knowledge representation, they lack the ability to understand or generate natural language. To achieve more natural and accurate human–machine interaction, the integration of Retrieval-Augmented Generation (RAG) is essential.

RAG is a technique that combines information retrieval with generative artificial intelligence to improve the factual accuracy and contextual relevance of generated content. Its core mechanism involves retrieving the most relevant textual passages from external knowledge sources based on a user query, and incorporating these passages—along with the original query—into the prompt provided to the LLM. The model then generates responses that are contextually grounded and verifiable [78].

Traditional RAG architectures often store external knowledge in flat, unstructured formats, which limits the model’s ability to capture complex relationships between entities. In contrast, the structured nature of KG helps eliminate irrelevant noise and enhances the language model’s capacity for semantic understanding and reasoning over user queries [79]. Furthermore, MAS can not only coordinate and optimize the retrieval and generation processes within the RAG framework, but also act as an executor of RAG-generated outputs—enabling downstream applications such as intelligent Q&A and personalized recommendation [80]. Studies have demonstrated that the integration of KG, RAG, and MAS can substantially mitigate hallucinations and enhance both the completeness and accuracy of generated responses [80]. In the subsequent sections, we provide a detailed explanation of the integration of RAG with MAS for knowledge extraction and intelligent task execution in the context of ACMC.

## Intelligent transmission of ACMC

Next, we will focus on the field of ACMC and explore how to overcome the limitations of traditional methods by combining KG and MAS technologies, thereby achieving the effective inheritance and innovative development of TCM knowledge. In response to the challenges mentioned above, we propose two approaches: first, using MAS to construct KG; and second, employing KG as the fundamental knowledge framework for MAS operations.

### Intelligence in KG extraction and construction

As we have described above, the intelligent construction of KG for ACMC faces significant technical barriers, primarily due to its unique characteristics, such as complex grammatical structures, a wealth of synonyms, and abstract TCM concepts. These features create multiple challenging technical tasks, which can be effectively distributed among different agents to collaboratively accomplish tasks such as translating ancient Chinese medical texts [81], synonym recognition [82], automated extraction [83], and KG completion [84].

In MAS design, integrating advanced NLP technologies has become an important trend in current MAS research. For example, Rui Yang [85] designed a framework called Graphusion, which employs methods such as BERTopic, LLM, and graph neural networks, and performs well in entity extraction and relationship recognition. Similarly, we can consider integrating traditional NLP or machine learning algorithms with LLMs, leveraging the strengths of each to continuously optimize extraction strategies and improve the efficiency and quality of KG construction. In this context, LLMs such as Qibo, MedChatZH, Huang-Di, and Lingdan—which incorporate ACMC as part of their training data—as well as the iFLYTEK Spark Cognitive Large Model, which has already achieved promising results in TCM, can all serve as reference tools for constructing MAS. Through this intelligent division of labor and collaboration, we aim to overcome the limitations of traditional methods in information integration and understanding, streamlining and enhancing the extraction of entities and relationships from classical ACMC.

Additionally, we propose that the first step should be to construct the ontology model for different ACMC because the effectiveness of LLM usage is highly dependent on the design of the Prompt. Here, “ontology” does not refer to the broad concepts in the field of TCM, but rather to the unique theoretical systems and diagnostic frameworks specific to each classical text. For example, *Treatise on Cold Damage* uses “Syndrome differentiation of six meridians” as the top levels of the hierarchy, which is further divided into sub-concepts like syndromes, treatment methods, and formulas, all interconnected through relations such as “belongs to” and “treat”, forming a complete theoretical framework. On the other hand, Item *Differentiation of Warm Febrile Diseases* is structured around “Wei-Qi-Ying-Blood” and “Syndrome differentiation of San Jiao” as its top-level concepts. Therefore, to accurately design prompts for LLMs tailored to ACMC, it is essential to deeply analyze the unique modes of thinking and diagnostic principles within each text. Based on this analysis, we can design prompts that will effectively trigger the LLM to accurately understand and generate professional TCM knowledge.

While ontology-based prompt engineering provides a crucial control interface for LLMs, enhancing adaptability—the ability to handle different textual styles and theoretical frameworks—and coverage—the comprehensiveness of extracted entities, attributes, and relations—is equally essential for achieving robust and complete knowledge extraction. To this end, we propose incorporating RAG within the MAS framework, allowing agents to retrieve and integrate external knowledge—both structured and unstructured—on demand. This enables dynamic construction, refinement, and extension of TCM knowledge graphs in complex semantic environments.

As shown in Table 3, we illustrate the respective roles of MAS and RAG in the intelligent construction of KG derived from the ACMC. For example, within the MAS-RAG framework, RAG modules play a crucial role in enhancing semantic fidelity during classical-to-modern Chinese translation and synonym resolution. While MAS coordinates the translation process and allocates agents to handle term normalization, RAG dynamically retrieves parallel expressions, historical commentaries, and dictionary definitions to support disambiguation and accurate rendering.

**Table 3:** Task allocation between MAS and RAG for knowledge graph construction

Subtasks of KG construction	MAS function	RAG enhancement
Classical text translation	Coordinate translation agent (to ensure terminology consistency and manage domain expert model scheduling)	Retrieve annotations and parallel corpora from classical texts to provide contextual references
Synonym recognition	Manage the entity normalization workflow and assign synonym aggregation tasks	Provide terminology usage examples and contextual evidence from multiple literature sources to support disambiguation
Entity recognition	Invoke named entity recognition agent to extract various entity types (e.g., Prescriptions names, Syndrome Name)	Retrieve relevant classical text passages to assist in resolving ambiguities (e.g., “Mahuang” as a plant or a prescription name)
Attribute extraction	Call attribute extraction agent to identify various attributes (e.g., medicinal properties, meridian tropism)	Retrieves entity-related descriptive texts to supply attribute extraction data sources
Relation extraction	Invoke relation identification agents to construct graph edges (e.g.,“Syndrome–Symptom” and “Prescription–Chinese Herbal Medicine), and verify logical consistency of relations	Provide literature evidence snippets supporting the existence of relations
Entity alignment/linking	Interfaces with existing KG terminology repositories to handle synonym/alias matching via dedicated agents	Leverages retrieved term contexts to enhance entity linking accuracy
Knowledge storage/updating	Perform version control and duplicate detection	Generate knowledge summaries with traceable citations

The integrated MAS-RAG framework demonstrates particular efficacy in processing non-standardized linguistic expressions characteristic of ACMC texts. Its core strengths lie in: (1) RAG dynamically retrieving multi-source classical references (e.g., commentaries, lexicons, parallel texts) to enhance the verifiability and semantic accuracy of task outputs; (2) MAS orchestrating domain-specific expert agents to ensure procedural rigor and system scalability. This collaborative mechanism helps enhance the procedural structure and semantic accuracy of KG construction from ACMC.

**Fig. 2:**
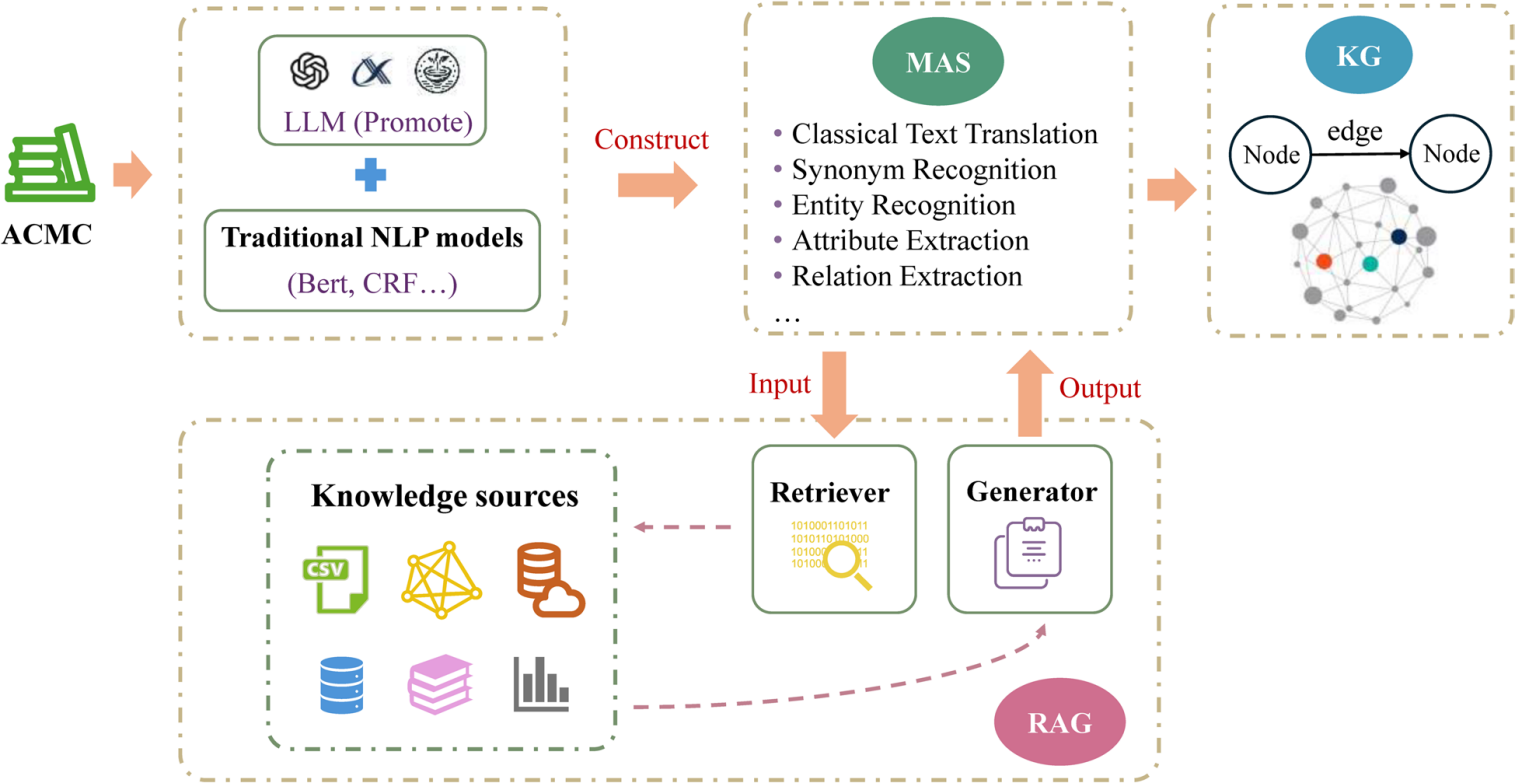
The intelligent workflow for KG construction from ACMC

Figure 2 illustrates the intelligent workflow for KG construction from ACMC. The integration of MAS and RAG enables collaborative coordination in task allocation, semantic enhancement, and knowledge generation, thereby significantly improving the efficiency and semantic quality of ACMC-based KG construction.

### Intelligent KG-based scenario applications

Building on the applications of MAS in various fields, we can consider adapting it for intelligent applications in the field of ACMC. For example, based on the KG constructed for each classical text, we could develop intelligent systems such as a Q&A system, personalized education system, and intelligent diagnosis and treatment system.

**Fig. 3:**
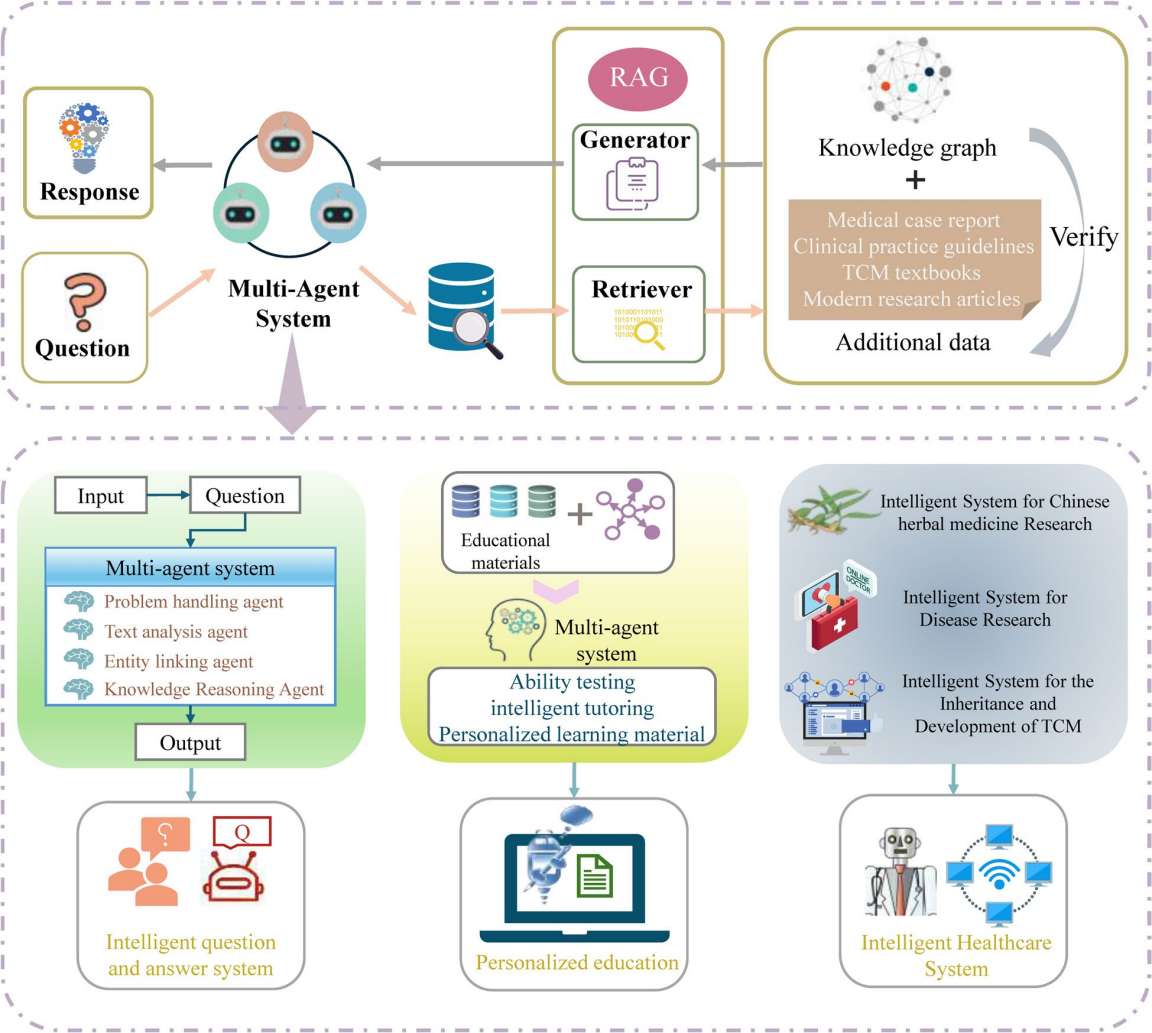
Schematic diagram of the intelligent inheritance pathway of ACMC

In this process, the RAG mechanism can still be introduced to enhance system performance. The retrieval component of RAG supports MAS from two directions [80]: first, by retrieving structured knowledge from the KG; second, by supplementing it with information from external sources such as Medical case reports, clinical practice guidelines, TCM textbooks, and modern research articles. The retrieved data are semantically validated through the KG and interpreted by the LLM before being delivered to the appropriate agents within the MAS. This facilitates the construction of a unified MAS framework in which different agents collaborate effectively based on KG/RAG integration. Figure 3 is the schematic diagram of the intelligent inheritance pathway of ACMC.

#### Design of intelligent Q&A systems

When building a Q&A system for the field of ACMC, tasks such as question identification, semantic segmentation, entity recognition, intent classification, classical text translation, synonym conversion, knowledge reasoning, and result output must be addressed. Current research trends are focusing on exploring how to integrate these individual tasks into a coherent, interdependent, and collaborative framework [86]. For example, Xinjie Zhao [87] proposed the AGENTi Graph framework, a MAS architecture, to address issues such as insufficient contextual understanding and flexibility, difficulty in querying nuanced or complex content, and the lack of seamless integration between KG and natural language interfaces in current Q&A systems. This framework enables dynamic interpretation of user intent, task management, and the integration of new knowledge. Therefore, we could consider incorporating this framework into the Q&A system for ACMC, adding agents designed specifically for classical text translation and synonym conversion to enhance the accuracy of the Q&A system for ACMC.

At the same time, in the evolution of knowledge retrieval paradigms, MAS provide a technical implementation framework for constructing dynamic collaborative retrieval pathways across heterogeneous data sources. For instance, the LLM-based dynamic MAS orchestration and retrieval framework proposed by Antony Seabra et al. [88] integrates RAG, Text-to-SQL conversion, dynamic prompt engineering, and distributed agent architectures. This framework not only resolves cross-modal data association challenges in complex query scenarios but also implements dynamic task allocation mechanisms while validating resource integration capabilities between unstructured documents (e.g., PDFs) and structured databases. These innovations offer methodological underpinnings for cross-source knowledge integration in TCM classics Q&A systems.

Given the deeply integrated nature of theoretical knowledge and clinical practice within ACMC, current KG exhibit significant limitations in representing nonlinear narrative knowledge such as historical physicians’ commentary annotations and clinical case records. Through multi-agent collaborative retrieval mechanisms, MAS transcend representational constraints of single knowledge sources, enabling cross-modal synergy among textual semantic parsing, structured data reasoning, and unstructured document mining. This hybrid retrieval paradigm enhances contextual relevance in answer generation while improving answer completeness through dynamic knowledge routing strategies, providing critical technical support for semantic generalization capabilities in TCM classics intelligent interpretation systems.

#### Design of intelligent educational systems

Similarly, the field of TCM education is actively seeking key approaches for intelligent integration, covering proficiency testing of medical students, assessment of cognitive levels and personalized recommendation of learning resources [89]. By introducing a hierarchical knowledge structure design based on KG, the efficiency of these tasks can be significantly enhanced [90]. On this solid foundation, we then construct some Agents for cognitive ability testing, intelligent tutoring, and personalized learning resource recommendations [91], aiming to build a MAS in the field of ACMC education. These Agents are not only highly intelligent but can also provide customized learning support according to medical students’ learning progress, interests, and knowledge levels. The cognitive ability testing Agent can accurately assess students’ mastery of TCM knowledge and cognitive abilities, providing scientific evidence for adjusting teaching plans. The intelligent tutoring Agent offers personalized learning guidance and Q&A services according to students’ specific needs, helping them overcome learning difficulties. Meanwhile, the personalized learning resource recommendation Agent makes full use of the rich resources from the “Four Classics of TCM”, and, by considering students’ individual characteristics and learning needs, precisely recommends relevant learning materials and case studies, further enhancing their learning interest and effectiveness. Research has also demonstrated that MAS-driven intelligent educational systems facilitate the development of adaptive learning models, enhancing learning outcomes [92]. Especially, for specialized educational content on tongue diagnosis in ACMC, integrating KG theories with image analysis agents can enhance students’ learning experience through more intuitive and explainable insights. This technique can draw on the framework proposed by Chenjun Li et al. [93], which integrates graph representation learning with vision-language models to deliver interpretable analytical results.

#### Design of an intelligent TCM healthcare system

In the design of intelligent TCM healthcare systems, we will elaborate on three dimensions: intelligent study on Chinese herbal medicine, intelligent investigation on diseases, and the intelligent inheritance and development of TCM.

##### Intelligent system for Chinese herbal medicine research

In the field of Chinese herbal medicine research, the core concerns for clinical application and drug development include the mechanisms of action (encompassing both TCM-theoretical mechanisms and modern multi-target pharmacological mechanisms), the principles of herbal compatibility, and the evaluation of safety and efficacy. KG enable the structured integration of these heterogeneous data sources, forming multidimensional semantic association networks—such as “Chinese herbal medicine–properties and flavors—meridian tropism—therapeutic functions—molecular targets”—thus providing both the data foundation and reasoning framework for the intelligent modeling and application of Chinese herbal medicine knowledge [94].

Building upon this foundation, a MAS for Chinese herbal medicine research can be constructed, comprising functional modules such as a classical literature efficacy analysis agent, a clinical compatibility agent, a modern pharmacological analysis agent, and a toxicity assessment agent. Through task-oriented collaboration and coordinated interaction among agents, the system can enable intelligent perception, semantic reasoning, and dynamic feedback across various stages of research, including mechanism analysis, comprehensive pharmacological evaluation, and clinical decision support.

For example, when a user initiates a query to analyze *Cinnamomi Ramulus*, the system can employ a RAG mechanism to jointly access and integrate information from the KG and external databases. The classical literature efficacy analysis agent can provide descriptions from historical texts. The modern pharmacological analysis agent can evaluate its multi-target mechanisms of action, pharmacokinetic and pharmacodynamic characteristics, and further infer potential therapeutic mechanisms based on ingredient–target interaction prediction models. The toxicity assessment agent can identify potentially toxic components, dosage thresholds, and risks associated with long-term use. Meanwhile, the clinical compatibility agent can supply information on commonly co-prescribed herbs and their corresponding disease or syndrome targets (e.g., the combination of *Cinnamomi Ramulus* and *Ephedra* is frequently used for treating allergic skin conditions [95]).

Building on the deep integration of LLM with KG, the proposed MAS system empowers agents with advanced functionalities such as cross-modal comprehension, semantic abstraction, dynamic task orchestration, and knowledge consistency verification. These capabilities facilitate sophisticated tasks including the structured fusion of classical and contemporary Chinese herbal medicine knowledge, multidimensional simulation of herbal compatibilities, reliable herbal combination recommendations, and the identification of potential pharmacodynamic mechanisms of Chinese herbal medicine.

This research paradigm has already demonstrated successful implementation in modern pharmacological studies. For instance, the DrugAgent system proposed by Yoshitaka Inoue et al. [96] utilizes multi-agent collaboration to predict complex drug—target interactions. The system integrates diverse data sources—including KG, biomedical literature, and kinase inhibitor databases—and deploys multiple specialized agents, such as an AI Agent, Search Agent, KG Agent, and Reasoning Agent. These agents collectively enable semantic understanding and reasoning over drug—target relationships, thereby significantly improving the robustness and accuracy of predictions in complex scenarios. This work provides a valuable methodological reference for designing analogous tasks within MAS-based systems for Chinese medicine.

##### Intelligent disease research system

Disease prevention, diagnosis, therapy, and rehabilitation represent fundamental pillars in the study of ACMC. The integration pathway between MAS and KG is structured around these dimensions, aiming to establish an intelligent framework that supports cognitive modeling, semantic reasoning, and practical application throughout the entire disease lifecycle.

First, in the domain of disease diagnosis and treatment, the implementation of an intelligent diagnostic and treatment system may require the integration of multiple agents, such as KG construction, symptom recognition, dynamic interaction, knowledge reasoning, complication inference, diagnostic decision-making, and feedback learning [97], to collaboratively complete tasks like structuring ACMC theories, supplementing symptom information, reasoning complex knowledge, structuring symptom data, inferring symptom-disease relationships, analyzing the connection between diseases and complications, making diagnostic judgments, optimizing model parameters, and improving the KG. Specifically, based on the KG of ACMC knowledge, structured data such as patients’ diagnostic summaries and prescription information from valid medical cases would be further supplemented, revealing the correlations between diseases and complications. Through dynamic interaction with the patient, the system can continuously update diagnostic information, adjust treatment plans, and proactively identify potential complications, leading to more accurate judgments and recommended treatment plans.

In recent years, the integration of TCM diagnostic and therapeutic principles with modern medical paradigms has demonstrated significant advantages in the management of various diseases. This synergistic effect has been validated by multiple multicenter clinical studies [98, 99]. Prescriptions based on syndrome differentiation and treatment have continuously expanded their application boundaries within modern medical systems, showing unique therapeutic value in the management of complex conditions such as ulcerative colitis [100], acute pharyngitis [101], and atopic dermatitis [102]. To move beyond the conventional paradigm of experiential medicine, Jiacheng Tang et al. [103] proposed a novel reasoning framework that integrates MAS with Chain-of-Thought methodologies. This framework aims to semantically decode the metaphorical language embedded in TCM theories and accurately map these concepts to corresponding pathophysiological entities in modern medicine through collaborative and interpretable reasoning processes. By constructing a dedicated KG, the study established structured associations between TCM metaphorical terminology and biomedical concepts from Western medicine. Furthermore, it introduced three core types of intelligent agents—TCM expert agents, Western medicine expert agents, and coordination agents—which engage in multi-round interactions to collaboratively perform integrated interpretive and reasoning tasks across both medical systems. This framework not only represents the first formalized modeling approach for the integration of TCM and Western medical theories but also provides a mechanism for transforming qualitative syndrome differentiation into quantitative, mechanistic reasoning for clinical decision support. As such, this work offers critical theoretical and technical insights for the design of MAS-based diagnostic systems, supporting the development of intelligent, interpretable, and interoperable models that integrate TCM diagnostic philosophy with modern biomedical knowledge. It also demonstrates strong potential for scalability and clinical translation.

Meanwhile, when constructing the relationship between diseases and complications, we can approach it from two perspectives. Taking *Treatise on Cold Damage* as an example, a misdiagnosed or unresolved Taiyang meridian disease could potentially lead to the combination of Taiyang and Yangming meridian diseases. On the other hand, with the development of integrative traditional Chinese and Western medicine, we can also apply the theory of disease progression in the six meridians to analyze potential complications of modern diseases [104]. This can help establish a comprehensive system for identifying complications through the integration of both medical approaches.

Secondly, in the domain of disease prevention, additional functional modules can be integrated into the aforementioned MAS framework, including agents for constitution identification and risk assessment, seasonal nodes guidance, symptom warning, preventive prescription recommendation, and lifestyle intervention. The knowledge base of these agents is primarily grounded in classical theoretical systems from the *The Yellow Emperor’s Classic of Medicine*, particularly those concerning constitution classification, seasonal health preservation, and the pathogenic mechanisms and preventive principles related to emotional and environmental factors [105]. Users may input multimodal information such as subjective symptoms and tongue images, enabling the constitution identification and risk assessment agent to determine their constitutional type and assess potential pre-disease states. By incorporating external risk factors—such as seasonal changes, geographical location, and emotional status—the system provides individualized evaluations of disease susceptibility. Subsequently, the relevant preventive agents are dynamically invoked to deliver tailored health-preserving recommendations, prescription suggestions, or lifestyle modification plans. In this way, the system enables early warning and proactive intervention in alignment with the TCM principle of treating disease before its onset.

Finally, an intelligent disease rehabilitation framework grounded in the integrative principles of ACMC can be further established. This framework aims to achieve individualized regulation, precise syndrome differentiation, dynamic intervention, and continuous evaluation of therapeutic outcomes. Unlike modern rehabilitation models, the ACMC system emphasizes a holistic philosophy of recovery—“Body-Spirit Cultivation”and “Healthy Qi Reinforcement and Pathogen Elimination”. It encompasses a wide range of TCM-specific modalities, including Daoist gymnastics, Qigong, acupuncture, therapeutic massage, herbal modulation via classical prescription, and dietary therapy [106]. Building upon this foundation, the intelligent disease research system may further incorporate a dedicated Rehabilitation Therapy Agent, which leverages diagnostic information integrated by the MAS-based diagnostic system to automatically assess and match the most appropriate rehabilitation strategies. This enables intelligent decision-making and personalized recommendations for TCM-based rehabilitation interventions.

##### Intelligent system for the inheritance and development of TCM

The core knowledge of ACMC is often embedded in unstructured carriers such as traditional cultural texts, classical medical literature, apprenticeship-based teaching, and clinical case records. These sources are characterized by ambiguous expressions, deeply metaphorical semantics, and poorly defined evolutionary trajectories, posing significant challenges to quantification and computational modeling [107]. Consequently, ensuring the effective inheritance and dynamic updating of this knowledge system has become a critical bottleneck hindering its continuous innovation and modern transformation. By constructing a collaborative knowledge evolution system that integrates KG, RAG, and MAS, it becomes possible to enable distributed coordination, semantic inference, and modular task execution, thereby facilitating the structured preservation and dynamic expansion of ACMC knowledge in the context of digital and intelligent transformation.

Specifically, based on the complexity of the aforementioned knowledge ecosystem, multiple thematic KG can be constructed, including ACMC, Cultural-Semantic, Scholastic Lineage, and Clinical Case. These graphs are designed to model and extract: (1) medical theories and treatment principles from ACMC; (2) philosophical concepts and metaphorical representations from traditional works such as the *I Ching* and *Book of Songs*; (3) academic systems from historical medical writings; and (4) diagnostic-treatment patterns and syndrome regularities from clinical case records. Within this framework, different graphs can be aligned at the entity level through a unified TCM Ontology, which enables term normalization, relation standardization, and semantic alignment. At the semantic level, this supports the formation of a multi-layered, multi-source interconnected knowledge network [108]. By incorporating relation types such as originates from, cites, evolves into, embodied in, and inferred as, the system can reveal logical chains and evolutionary pathways of TCM knowledge, thereby supporting bidirectional association and provenance analysis between theoretical knowledge and experiential evidence. Building upon this semantic infrastructure, the KG serves as the foundational knowledge base, while RAG provides a mechanism for knowledge enhancement. On this basis, several intelligent agents can be deployed, including a Classical Literature Parsing Agent, Apprenticeship Simulation Agent, Case Analysis Agent, and Knowledge Evolution Coordination Agent. These agents are responsible for semantic abstraction of ancient medical literature, modeling lineage-based knowledge transmission, mining clinical experience, and integrating cross-source knowledge, respectively.

Using the aforementioned MAS framework as an example, we aim to facilitate a paradigm shift in the TCM knowledge system—from fragmented experiential knowledge to systematic and evolving structures. This transformation aims to migrate metaphorical and abstract medical concepts into computable and interpretable semantic representations, thereby enabling a transition from the traditional apprenticeship-based oral transmission model to a new path characterized by KG–driven collaborative construction and intelligent evolution.

Similarly, the deep integration of KG and MAS opens up new technological pathways for the theoretical innovation and systematic development of the ACMC framework. This integration can be reflected in several dimensions, including cross-paradigm reconstruction of theoretical systems, emergent generation of prescription, and genealogical modeling of academic schools.

First, in contrast to the relatively well-preserved system of TCM transmission, the development of its foundational theories has lagged behind in recent decades. Since 1949, most related studies have focused on the collation and interpretation of traditional doctrines, with few major theoretical breakthroughs, highlighting the urgent need to stimulate the field’s overall innovative potential [109]. Previous research has suggested that the theoretical foundations of TCM are deeply rooted in a multidisciplinary cultural system of ancient China, encompassing philosophy, astronomy, mathematics, and other domains. This intrinsic interdisciplinarity provides important clues for theoretical innovation in TCM [110]. Therefore, constructing a heterogeneous KG that integrates TCM with other classical disciplinary knowledge provides a solid foundation for theoretical innovation. On this basis, multiple intelligent agents can be deployed to support cross-domain knowledge interpretation, integrative reasoning of TCM theories, and coordinated theoretical evolution, thereby promoting systematic advances in fundamental TCM theory.

Secondly, with the introduction of modern medicine into China, the innovation of prescription has not only continued the classical principles of formulation (Monarch-Minister-Assistant-Envoy), but has also gradually incorporated findings from modern pharmacological research. This has led to a new model that integrates theoretical deduction with empirical evidence. A representative example is the development of *Gastrodiae-Uncariae Decoction* for the treatment of hypertension by Guangci Hu [111]. Inspired by this approach, an intelligent prescription innovation system can be established by integrating KG with MAS. This system would aggregate heterogeneous information from multiple sources, including ACMC-based prescription knowledge, prescription annotations, clinical case records, and modern pharmacological research. Core agents can be designed and deployed to support specific tasks, including formula-syndrome structure modeling, extraction of prescription principles, pharmacological validation, reverse reasoning from syndrome to Chinese herbal medicine, Chinese herbal medicine interaction and risk assessment, and coordinated innovation management. Through semantic reasoning and collaborative modeling, these agents work together to support the full-cycle generation of new prescriptions, from theoretical logic and experiential inheritance to modern empirical validation. In doing so, the system facilitates a shift from experience-driven prescription practices to a computable, interpretable, and systematized innovation pathway in Chinese medicine.

Finally, numerous academic schools have emerged from the theoretical foundation of ACMC, each with its own distinctive theoretical emphasis and practical framework. However, significant heterogeneity among these schools has posed challenges for theoretical integration and systematization [112]. To address this issue, KG technologies can be employed to construct semantic tree structures of multi-school therapeutic approaches, using representative syndromes as anchor nodes. Under the integrative framework of Disease-Pulse Diagnosis-Syndrome Pattern-Treatment, multidimensional edge types such as inheritance, integration, and evolution can be design to systematically map the theoretical relationships and academic trajectories across different schools. Furthermore, a MAS can be designed and deployed to support this process, comprising modules such as a School Knowledge Parsing Agent, School Similarity and Difference Analysis Agent, Integration Reasoning Agent, Academic Evolution Detection Agent, and Innovation Strategy Design Agent. Together, these agents form a multi-source knowledge modeling and reasoning system for academic schools of TCM, offering methodological support for exploring school-based innovation pathways.

### Possible challenges

Although the integration of MAS and KG is considered an effective approach for the intelligent development of ACMC, it still faces multiple challenges in practical application. First, the reasoning process of ACMC knowledge is heavily influenced by professional barriers, with many causal relationships between knowledge points relying on experiential transmission. This increases the difficulty of accurately converting these causal relationships into quantifiable models and data, further challenging the accuracy and practicality of intelligent systems. Second, users typically expect the system to provide clear reasoning processes and explanations for its results. However, MAS and KG-driven models are often seen as black boxes, which may not offer satisfactory explanations. This lack of transparency severely undermines the interpretability and reliability of intelligent applications, thereby affecting their acceptance and effectiveness in clinical settings. Therefore, the goal of intelligent development for ACMC may be more focused on serving as an auxiliary tool, particularly in the field of intelligent diagnosis, where it is still unable to fully replace the role of human experts.

## Conclusion

The intelligent development of ACMC is entering a new phase, with KG becoming a key research area. However, challenges remain in the intelligent construction and application of KG. The integration of KG and MAS technologies showcases tremendous potential for the intelligent inheritance of ACMC knowledge. Through intelligent collaboration and division of labor, it breaks traditional limitations and enhances the efficiency of entity and relationship extraction from ACMC texts. Meanwhile, intelligent applications based on KG, such as intelligent Q&A systems, educational platforms, and diagnostic systems, bring innovation and development to the TCM. Although this convergence shows great potential, it may still have to face multiple challenges such as professional challenges in knowledge reasoning and lack of system interpretability. Therefore, it is evident that the intelligent development of ACMC still holds vast potential for exploration and optimization.

## Data Availability

Not applicable.

## Abbreviations

ACMC: Ancient Chinese Medicine Classics
KG: Knowledge graphs
Q&A: Question-answering
LLMs: Large language models
MAS: Multi-agent systems
TCM: Traditional Chinese Medicine
AI: Artificial intelligence
NLP: Natural language processing
RAG: Retrieval-augmented generation

## References

[1] Huang K, Zhang P, Zhang Z, et al. Traditional Chinese medicine (tcm) in the treatment of covid-19 and other viral infections: efficacies and mechanisms. Pharmacol Ther. 2021;225:107843. 33811957 10.1016/j.pharmthera.2021.107843 PMC8011334

[2] Ye X, Dong MH. A review on different English versions of an ancient classic of Chinese medicine: Huang di nei jing. J Integr Med. 2017;15:11–8. 28088255 10.1016/S2095-4964(17)60310-8

[3] Zhou X, Yang Q, Bi L, et al. Integrating traditional apprenticeship and modern educational approaches in traditional Chinese medicine education. Med Teach. 2024;46:792–807. 38052086 10.1080/0142159X.2023.2284661

[4] Zhou Y, Qi X, Huang Y, et al. Research on construction and application of tcm knowledge graph based on ancient chinese texts. IEEE/WIC/ACM International Conference on Web Intelligence-Companion Volume. 2019.

[5] Zhang S, Wang W, Pi X, et al. Advances in the application of traditional Chinese medicine using artificial intelligence: a review. Am J Chin Med. 2023;51:1067–83. 37417927 10.1142/S0192415X23500490

[6] Lin R, Wang B, Zhang N, et al. Study on knowledge map of ancient prescription books of tcm. 2019 10th International Conference on Information Technology in Medicine and Education (ITME). IEEE. 2019.

[7] Yu T, Li J, Yu Q, et al. Knowledge graph for tcm health preservation: design, construction, and applications. Artif Intell Med. 2017;77:48–52. 28545611 10.1016/j.artmed.2017.04.001

[8] Yang P, Wang H, Huang Y, et al. Lmkg: a large-scale and multi-source medical knowledge graph for intelligent medicine applications. Knowl-Based Syst. 2024;284:111323.

[9] Peng C, Xia F, Naseriparsa M, et al. Knowledge graphs: opportunities and challenges. Artif Intell Rev. 2023;56:13071–102. 10.1007/s10462-023-10465-9 PMC10068207 37362886

[10] Li S, Li Z, Xue K, et al. Gc-cdss: personalized gastric cancer treatment recommendations system based on knowledge graph. Int J Med Inform. 2024;185:105402. 38467099 10.1016/j.ijmedinf.2024.105402

[11] ALMutairi M, AlKulaib L, Wang S, et al. Fhirviz: Multi-agent platform for fhir visualization to advance healthcare analytics. Proceedings of the 15th ACM International Conference on Bioinformatics, Computational Biology and Health Informatics. 2024.

[12] Dongbo L, Changfa W, Shuaishuai X, et al. Construction and application of knowledge graph of treatise on febrile diseases. Digit Chin Med. 2022;5:394–405.

[13] Feng Y. Research on knowledge extraction technology for the yellow emperor’s classic of medicin. Master’s thesis. Tangshan, Hebei: North China University of Science and Technology; 2023.

[14] Fensel D, Şimşek U, Angele K, et al. Introduction: What is a knowledge graph? In: Knowledge graphs. Cham: Springer; 2020. p. 1–10.

[15] Zhou H, Shen T, Liu X, et al. Survey of knowledge graph approaches and applications. J Artif Intell. 2020;2:89–101.

[16] Qu K, Li KC, Wong BT, et al. A survey of knowledge graph approaches and applications in education. Electronics. 2024;13:2537.

[17] Fensel D, Şimşek U, Angele K, et al. How to build a knowledge graph. In: Knowledge graphs. Cham: Springer; 2020. p. 11–68.

[18] Chen Z, Wang Y, Zhao B, et al. Knowledge graph completion: a review. IEEE Access. 2020;8:192435–56.

[19] Wu Y, Jiao N, Li X, et al. Correlation analysis between scores of grade examination of TCM classics competence and scores of TCM classics courses. Educ Chin Med. 2022;41:22–6.

[20] Wang S, Zhang S, Ma Y. Enhancing pedagogical approaches to classical TCM texts for cultivating Competent clinical practitioners. Hunan J Tradit Chin Med. 2018;34:125–6.

[21] Li Q, Fu X, Yang F, et al. Construction and application of a knowledge graph of the treatise on cold damage based on the diagnostic thinking of “disease, pulse, symptom, and treatment.” Mod Trad Chin Med Materia Med-World Sci Technol. 2022;24:3613–21.

[22] Yang F, Zhong X, Zhou R, et al. Research on the annotation scheme and knowledge representation oftreatise on febrile and miscellaneous diseases based on knowledge unit theory. Chin Med Herald. 2022;19:12–6.

[23] Zhang Y, Sun Y, Li Y. Research on the construction of the “treatise on cold damage” ontology. Chin J Chin Med. 2024;39:1841–6.

[24] Kuang H. Construction of a knowledge graph for the treatise on cold damage and its application in yangming disease. PhD thesis. Hangzhou, Zhejiang: Zhejiang Chinese Medical University; 2021.

[25] Qu Q. Research on the treatise on cold damage based on natural language processing. Master’s thesis. Hefei, Anhui: Anhui University of Chinese Medicine; 2021.

[26] Wang J. Research on an intelligent question-answering system for the treatise on cold damage based on knowledge graph. Master’s thesis. Changsha, Hunan: Hunan University of Chinese Medicine; 2022.

[27] Chu L, Tao T, Chen G, et al. A learning resource recommendation model for treatise on cold damage courses based on knowledge graphs. Mod Inf Technol. 2024;8:182–8.

[28] Zhang X. Construction and application research of knowledge graph for synopsis of prescriptions of the golden chamber. Master’s thesis. Changchun, Jilin: Jilin University; 2023.

[29] Zou X. Construction and application of knowledge graph of ancient books of epidemic febrile diseases school. Master’s thesis. Changchun, Jilin: Changchun University of Chinese Medicine; 2023.

[30] Bing W. Warm disease theory: Wen bing xue. Colorado: Paradigm Publications; 2003.

[31] Al-Moslmi T, Ocaña MG, Opdahl AL, et al. Named entity extraction for knowledge graphs: a literature overview. IEEE Access. 2020;8:32862–81.

[32] Qu X, Tian Z, Cui J, et al. A review of knowledge graph in traditional Chinese medicine: analysis, construction, application and prospects. Comput Mater Contin. 2024;81:3583.

[33] Liu Z, Peng E, Yan S, et al. T-know: A knowledge graph-based question answering and infor-mation retrieval system for traditional chinese medicine. Proceedings of the 27th international conference on computational linguistics: system demonstrations. 2018.

[34] Li X, Ren J, Zhang W, et al. Ltm-tcm: a comprehensive database for the linking of traditional Chinese medicine with modern medicine at molecular and phenotypic levels. Pharmacol Res. 2022;178:106185. 35306140 10.1016/j.phrs.2022.106185

[35] Chen X, Chen H, Bi X, et al. Biotcm-se: a semantic search engine for the information retrieval of modern biology and traditional chinese medicine. Comput Math Methods Med. 2014;2014:957231. 24772189 10.1155/2014/957231 PMC3989774

[36] Gao L, Jia C-H, Wang W. Recent advances in the study of ancient books on traditional Chinese medicine. World J Tradit Chin Med. 2020;6:61–6.

[37] Zhou R, Xing C, Li Q, et al. Knowledge unit indexing and graph analytics for the “disease-pulse-syndrome-treatment” framework in records of classical formula experiments. J Basic Chin Med. 2024;30:1210–5.

[38] Wang S, Sun X, Teng G, et al. Application of knowledge graph in multidimensional analysis of domain knowledge. Digit Libr Forum. 2019;3:18–27.

[39] Ye D, Mou L, Xu X. Interactive retrieval of traditional Chinese medicine prescriptions based on knowledge graphs. Digital Libr Forum. 2024;20:24–33.

[40] Liu H, Qu X, Shen Y, et al. Research on knowledge graph reasoning in traditional Chinese medicine. Mod Tradit Chin Med Mater Med-World Sci Technol. 2025;27:601–11.

[41] Cui M, Jia L, Yu T, et al. Current status of traditional Chinese medicine language system. Frontier and Future Development of Information Technology in Medicine and Education: ITME 2013. Springer. 2014.

[42] Ma Y, Feng Y. Research on implicit relation extraction methods for traditional Chinese medicine ancient books. J Zhengzhou Univ. 2024;56:34–42.

[43] Hu S. An analysis of the linguistic artistry in the treatise on cold damage. Huaxia Cult Forum. 2022;2:252–61.

[44] Jia Q, Zhang D, Yang S, et al. Traditional Chinese medicine symptom normalization approach leveraging hierarchical semantic information and text matching with attention mechanism. J Biomed Inform. 2021;116:103718. 33631381 10.1016/j.jbi.2021.103718

[45] Wu Z, Liu Y, Huo Q, et al. Named entity recognition of tcm classics based on sikubert and multi-feature embedding. 2023 3rd International Conference on Consumer Electronics and Computer Engineering (ICCECE). IEEE. 2023.

[46] Krishna Kommineni V, König-Ries B, Samuel S. From human experts to machines: an LLM supported approach to ontology and knowledge graph construction. arXiv e-prints : arXiv: 240308345. 2024. 10.48550/arXiv.2403.08345.

[47] He Y, Li M, Li M, et al. Research on traditional chinese medicine knowledge graph entity and relationship extraction based on gpts. Shanghai J Trad Chin Med. 2024;58:1–6.

[48] Zhang Y, Hao Y. Traditional Chinese medicine knowledge graph construction based on large language models. Electronics. 2024;13:1395.

[49] Chen H, Shen X, Lv Q, et al. Sac-kg: exploiting large language models as skilled automatic constructors for domain knowledge graphs. arXiv preprint arXiv:241002811. 2024. 10.48550/arXiv.2410.02811.

[50] Grabb D. The impact of prompt engineering in large language model performance: a psychiatric example. J Med Artif Intell. 2023;6:1–5.

[51] Zhang H, Wang X, Meng Z, et al. Qibo: a large language model for traditional Chinese medicine. arXiv preprint arXiv:240316056. 2024. 10.48550/arXiv.2403.16056.

[52] Tan Y, Zhang Z, Li M, et al. Medchatzh: a tuning LLM for traditional Chinese medicine consultations. Comput Biol Med. 2024;172:108290. 38503097 10.1016/j.compbiomed.2024.108290

[53] Zhang J, Yang S, Liu J, et al. AIGC empowering the revitalization of ancient books on traditional Chinese medicine: building the Huang-Di large language model. Libr Trib. 2024;44:103–12.

[54] Hua R, Dong X, Wei Y, et al. Lingdan: enhancing encoding of traditional Chinese medicine knowledge for clinical reasoning tasks with large language models. J Am Med Inform Assoc. 2024;31:2019–29. 39038795 10.1093/jamia/ocae087 PMC11339528

[55] Zeng X, Tu X, Liu Y, et al. Toward better drug discovery with knowledge graph. Curr Opin Struct Biol. 2022;72:114–26. 34649044 10.1016/j.sbi.2021.09.003

[56] Liu J, Zhuo HH, Jin K, et al. Sequential condition evolved interaction knowledge graph for traditional Chinese medicine recommendation. arXiv preprint arXiv:230517866. 2023. 10.48550/arXiv.2305.17866.

[57] Elsahar H, Gravier C, Laforest F. Zero-shot question generation from knowledge graphs for unseen predicates and entity types. arXiv preprint arXiv:180206842. 2018. 10.48550/arXiv.1802.06842.

[58] Weizhou W, Xilan Z, Qi G, et al. Study on the adaptive test modelling based on knowledge graph in the domain of substation. J Phys: Conf Ser. 2022. 10.1088/1742-6596/2213/1/012017.

[59] Shi Y, Yue F. Metaphorical thinking of traditional Chinese medicine and its features. World J Tradit Chin Med. 2024;10:535–47.

[60] Jiang M, Lu C, Zhang C, et al. Syndrome differentiation in modern research of traditional Chinese medicine. J Ethnopharmacol. 2012;140:634–42. 22322251 10.1016/j.jep.2012.01.033

[61] Wang H, Wang D-F, Song H-X, et al. Discussion on the composing principle of" Qingfei Paidu decoction" in the treatment of anti-covid-19 from the theory of syndrome differentiation of the six meridians in treatise on febrile diseases. J Hainan Med Univ. 2020;19:1–6.

[62] Han J-Y, Li Q, Pan C-S, et al. Progression of the wei-qi-ying-xue syndrome, microcirculatory disturbances, in infectious diseases and treatment with traditional Chinese medicine. World J Tradit Chin Med. 2022;8:169–80.

[63] Liu X, Mao T, Shi Y, et al. Overview of knowledge reasoning for knowledge graph. Neurocomputing. 2024. 10.1016/j.neucom.2024.127571.

[64] Cheng Z, Chen X, Wang J, et al. A knowledge graph-based retrieval enhanced generative intelligence question and answer technique. Comp Sci. 2025;52:1–12.

[65] Dorri A, Kanhere SS, Jurdak R. Multi-agent systems: a survey. IEEE Access. 2018;6:28573–93.

[66] Amirkhani A, Barshooi AH. Consensus in multi-agent systems: a review. Artif Intell Rev. 2022;55:3897–935.

[67] Le TA, Jivalagian A, Hiba T, et al. Multi-agent systems and cancer pain management. Curr Pain Headache Rep. 2023;27:379–86. 37382870 10.1007/s11916-023-01131-4

[68] Aman B, Ciobanu G. Formal analysis of medical systems using multi-agent systems with information sharing. Comput Sci J Moldova. 2024;94:3–18.

[69] Pałka P, Olszewski R, Kęsik-Brodacka M, et al. Using multiagent modeling to forecast the spatiotemporal development of the covid-19 pandemic in Poland. Sci Rep. 2022;12:11314. 35789191 10.1038/s41598-022-15605-9 PMC9252566

[70] Alsassa S, Lefèvre T, Laugier V, et al. Modeling early stages of bone and joint infections dynamics in humans: a multi-agent, multi-system based model. Front Mol Biosci. 2020;7:26. 32226790 10.3389/fmolb.2020.00026 PMC7080862

[71] Zhang Q, Dong J, Chen H, et al. Knowgpt: knowledge graph based prompting for large language models. The Thirty-eighth Annual Conference on Neural Information Processing Systems. 2024.

[72] Deng Y, Xia M, Cao M, et al. Kgsms: knowledge graph sample based multi-agent simulation. 2022 IEEE 2nd International Conference on Electronic Technology, Communication and Information (ICETCI), IEEE. 2022.

[73] Lavendelis E. A cloud based knowledge structure update and machine learning framework for heterogeneous multi-agent systems. Int J Artif Intell. 2016;14:157–70.

[74] Ghafarollahi A, Buehler MJ. Sciagents: automating scientific discovery through bioinspired multi-agent intelligent graph reasoning. Adv Mater. 2025;37(22):2413523. 39696898 10.1002/adma.202413523 PMC12138853

[75] Zhao J, Liu X. Tcaf: a multi-agent approach of thought chain for retrieval augmented generation. 2024 KDD Cup Workshop for Retrieval Augmented Generation. 2024.

[76] Zhao R, Tang J, Zeng W, et al. Zero-shot knowledge graph question generation via multi-agent LLMs and small models synthesis. Proceedings of the 33rd ACM International Conference on Information and Knowledge Management. 2024.

[77] Li X, Shen Y, Chen L. Mcore: multi-agent collaborative learning for knowledge-graph-enhanced recommendation. 2021 IEEE International Conference on Data Mining (ICDM). IEEE. 2021.

[78] Lewis P, Perez E, Piktus A, et al. Retrieval-augmented generation for knowledge-intensive nlp tasks. Adv Neur Inf Process Syst. 2020;33:9459–74.

[79] Chen R. Retrieval-augmented generation with knowledge graphs: a survey. Computer Science Undergradaute Conference 2025@ XJTU. 2025.

[80] Yu HQ, McQuade F. Rag-kg-il: a multi-agent hybrid framework for reducing hallucinations and enhancing LLM reasoning through rag and incremental knowledge graph learning integration. arXiv preprint arXiv:250313514. 2025. 10.48550/arXiv.2503.13514.

[81] Zuo L, Zhao Z, Wang D. Research on automatic classification of siku quanshu based on large language model. J Inf Resour Manag. 2024;14:22–35.

[82] Wei J, Zou K. Eda: easy data augmentation techniques for boosting performance on text classification tasks. arXiv preprint arXiv:190111196. 2019. 10.48550/arXiv.1901.11196.

[83] Scrocca M, Celino I. Knowledge graph extraction from text with large language models. Knowledge Engineering and Knowledge Management: 24th International Conference, EKAW 2024, Amsterdam, The Netherlands, November 26–28. Proceedings. Springer Nature. 2024

[84] Wei Y, Huang Q, Kwok JT, et al. Kicgpt: large language model with knowledge in context for knowledge graph completion. arXiv preprint arXiv:240202389. 2024. 10.48550/arXiv.2402.02389.

[85] Yang R, Yang B, Ouyang S, et al. Graphusion: leveraging large language models for scientific knowledge graph fusion and construction in nlp education. arXiv preprint arXiv:240710794. 2024. 10.48550/arXiv.2407.10794.

[86] Li R, Ren G, Yan J, et al. Intelligent question answering system for traditional Chinese medicine based on BSG deep learning model: taking prescription and Chinese materia medica as examples. Digital Chin Med. 2024;7:47–55.

[87] Zhao X, Blum M, Yang R, et al. Agentigraph: an interactive knowledge graph platform for LLM-based chatbots utilizing private data. arXiv preprint arXiv:241011531. 2024. 10.48550/arXiv.2410.11531.

[88] Seabra A, Cavalcante C, Nepomuceno J, et al. Dynamic multi-agent orchestration and retrieval for multi-source question-answer systems using large language models. Int J on Cybernetics & Informatics. 2024. 10.5121/ijci.2024.130602.

[89] Yang L, Wang Z, Yao K, et al. Prospective reflections on the application of big language modelling in traditional Chinese medicine. Chin J Trad Chin Med. 2025;43:1–20.

[90] Qin Y, Gao C, Wei S, et al. Learning from hierarchical structure of knowledge graph for recommendation. ACM Trans Inf Syst. 2023;42:1–24.

[91] Razafinirina MA, Dimbisoa WG, Mahatody T. Pedagogical alignment of large language models (LLM) for personalized learning: a survey, trends and challenges. J Intell Learn Syst Appl. 2024;16:448–80.

[92] Wu H-M, Yin T, Chan Y-J. Using a conversation-based agent system to foster math argumentation learning. Educ Technol Res Dev. 2025. 10.1007/s11423-025-10455-4.

[93] Li C, Lux L, Berger AH, et al. Fine-tuning vision language models with graph-based knowledge for explainable medical image analysis. arXiv preprint arXiv:250309808. 2025. 10.48550/arXiv.2503.09808.

[94] Duan P, Yang K, Su X, et al. Htinet2: herb–target prediction via knowledge graph embedding and residual-like graph neural network. Brief Bioinform. 2024;25:bbae414. 39175133 10.1093/bib/bbae414 PMC11341278

[95] Fan YP, Xiong XJ. Chinese classical formulas ephedra associated prescriptions for treatment of skin diseases. Chin J Chin Mater Med. 2018;43:2431–4. 10.19540/j.cnki.cjcmm.2018.0074 29950055

[96] Inoue Y, Song T, Wang X, et al. Drugagent: multi-agent large language model-based reasoning for drug-target interaction prediction. ICLR 2025 Workshop on Machine Learning for Genomics Explorations. 2025.

[97] Li J, Li Q, Guo Z, et al. Intelligent diagnosis and treatment system based on medical knowledge graph. 2024 6th International Conference on Data-driven Optimization of Complex Systems (DOCS). IEEE. 2024.

[98] Liu M, Gao Y, Yuan Y, et al. Efficacy and safety of integrated traditional Chinese and western medicine for corona virus disease 2019 (COVID-19): a systematic review and meta-analysis. Pharmacol Res. 2020;158:104896. 32438037 10.1016/j.phrs.2020.104896 PMC7211759

[99] Li CY, Ain Mohd Tahir N, Li SC. A systematic review of integrated traditional Chinese and western medicine for managing irritable bowel syndrome. Am J Chin Med. 2015;43:385–406. 25916468 10.1142/S0192415X15500251

[100] Wang Y, Zhang J, Zhang B, et al. Modified Gegen Qinlian decoction ameliorated ulcerative colitis by attenuating inflammation and oxidative stress and enhancing intestinal barrier function in vivo and in vitro. J Ethnopharmacol. 2023;313:116538. 37086872 10.1016/j.jep.2023.116538

[101] Huang H, Wu D, Li Q, et al. Jiegeng decoction ameliorated acute pharyngitis through suppressing nf-κb and mapk signaling pathways. J Ethnopharmacol. 2024;332:118328. 38734391 10.1016/j.jep.2024.118328

[102] Yuan H, Tang Y, Zhang S, et al. Nlrp3 neuroinflammatory intervention of mahuang-lianqiao-chixiaodou decoction for mental disorders in atopic dermatitis mice. J Ethnopharmacol. 2024;319:117263. 37783411 10.1016/j.jep.2023.117263

[103] Tang J, Wu N, Gao F, et al. From metaphor to mechanism: how LLMs decode traditional Chinese medicine symbolic language for modern clinical relevance. arXiv preprint arXiv:250302760. 2025. 10.48550/arXiv.2503.02760.

[104] Zhao X. Exploration of Chinese and western convergence on the six meridians in the treatment of type 2 diabetes mellitus and its complications. 2021.

[105] Lou X, Bai M, Miao M-S. An overview and modern discussion on the folk custom of keeping health in the eastern four seasons. 3rd Annual 2017 International Conference on Management Science and Engineering (MSE 2017). Atlantis Press. 2017.

[106] Sun Y, Han S, Y J. Exploring the theory and method of rehabilitation of traditional chinese medicine from ancient books of traditional Chinese medicine. Beijing J Tradit Chin Med. 2019;38:1121–5.

[107] Zhu B, Xu J, Shen J. Research on the cultural origin, inheritance model and path of tacit knowledge of Chinese medicine: based on the perspective of distributed cognition. Med Philos. 2018;39:78–82.

[108] He Y, Chen J, Antonyrajah D, et al. Bertmap: a bert-based ontology alignment system. Proceedings of the AAAI Conference on Artificial Intelligence. 2022.

[109] Xue X, Liu C, Wang Y, et al. Status quo and development strategy of traditional Chinese medicine in the new era. Strateg Stud CAE. 2023;25:78–82.

[110] Wei W, Zhang Q. Direction of innovation and development of traditional Chinese medicine theory from the perspective of cultural attributes. J Tradit Chin Med. 2020;61:185–8.

[111] Xiong Q, Li Y, Shen Y, et al. An analytical study of Hu Guangci’s new insights into the diagnosis and treatment of miscellaneous internal diseases in traditional Chinese medicine from the Sichuan–Chongqing region. Tradit Chin Med Res. 2023;36:69–73.

[112] Zheng H. Research on academic schools of Chinese medicine from the perspective of medium. J Nanjing Univ Tradit Chin Med. 2022;23:361–4.

